# Bayesian module identification from multiple noisy networks

**DOI:** 10.1186/s13637-016-0038-9

**Published:** 2016-02-05

**Authors:** Siamak Zamani Dadaneh, Xiaoning Qian

**Affiliations:** Department of Electrical and Computer Engineering, Texas A&M University, MS 3128, TAMU, College Station, TX USA

**Keywords:** Module identification, Stochastic block model, Multiple-network clustering, Bayesian clustering, Variational Bayes algorithm

## Abstract

**Background and motivations:**

Module identification has been studied extensively in order to gain deeper understanding of complex systems, such as social networks as well as biological networks. Modules are often defined as groups of vertices in these networks that are topologically cohesive with similar interaction patterns with the rest of the vertices. Most of the existing module identification algorithms assume that the given networks are faithfully measured without errors. However, in many real-world applications, for example, when analyzing protein-protein interaction networks from high-throughput profiling techniques, there is significant noise with both false positive and missing links between vertices. In this paper, we propose a new model for more robust module identification by taking advantage of multiple observed networks with significant noise so that signals in multiple networks can be strengthened and help improve the solution quality by combining information from various sources.

**Methods:**

We adopt a hierarchical Bayesian model to integrate multiple noisy snapshots that capture the underlying modular structure of the networks under study. By introducing a latent root assignment matrix and its relations to instantaneous module assignments in all the observed networks to capture the underlying modular structure and combine information across multiple networks, an efficient variational Bayes algorithm can be derived to accurately and robustly identify the underlying modules from multiple noisy networks.

**Results:**

Experiments on synthetic and protein-protein interaction data sets show that our proposed model enhances both the accuracy and resolution in detecting cohesive modules, and it is less vulnerable to noise in the observed data. In addition, it shows higher power in predicting missing edges compared to individual-network methods.

## Introduction

Identifying modular structures within large-scale networks has attracted significant attention in many research fields, including social science, biology, and information technology, just to name a few. For these applications, the ultimate goal is to group vertices in given networks into cohesive modules or communities, in which the vertices share similar properties, specifically their interaction patterns. Typically, densely connected sub-networks in given networks are considered desirable modular structures [1]. There have been many existing approaches proposed to study this problem in the literature, including spectral clustering algorithms based on graph cut [2, 3], modularity-based algorithms [4, 5], as well as matrix factorization algorithms for network clustering [6, 7].

In addition to these optimization algorithms based on graph theory and mathematical programming, in statistical inference, stochastic block models (SBM) originally proposed by [8] adopt a multinomial-Bernoulli probabilistic model to capture the inherent modular structures in observed networks. Hofman and Wiggins [9] developed a Bayesian framework to find the module or community memberships of vertices in networks under study and took advantage of variational approximation to efficiently sample from the corresponding posterior distributions.

Extending the analysis to dynamic networks has attracted major attention recently. Authors in [10] studied community evolution in blogosphere based on graph characteristics such as in-degrees and out-degrees. Chi et al. [11] used graph cut size as a measure of community evolution and proposed a dynamic version of spectral clustering. In [12], an algorithm called FacetNet was developed by extending the graph factorization method for analysis of evolutionary networks. A Markov model [13] was adopted to capture temporal community variation in stochastic block models with Gibbs sampling implemented for inference of unknown model parameters.

**Fig. 1:**
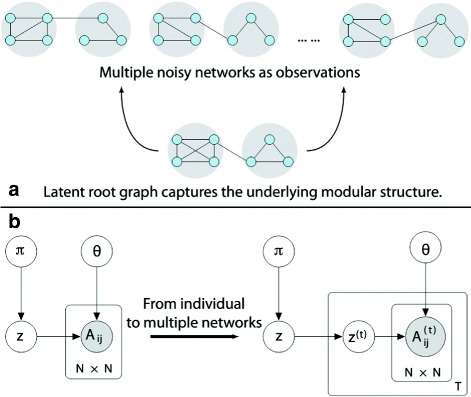
Schematic illustration. **a** An example of multiple noisy networks with a coherent modular structure. **b** Graphical representation of the proposed probabilistic model for module identification across multiple networks as an extension of the module identification model for individual networks

In this paper, we focus on module identification in biological networks. On one hand, it is often the case that biological networks in public databases [14, 15], by either high-throughput profiling techniques or laborious manual curations, contain significant errors (both false positives and false negatives). On the other hand, usually, several independent such empirical networks are available for studying the species of interest, creating the opportunity to integrate information from different sources and gain higher accuracy and better reproducibility. With these noisy networks, we aim to develop an integrated stochastic model and solution methods to improve the accuracy of module identification by combining information from multiple observed networks. Figure 1 provides the schematic illustration of our basic idea. With multiple noisy observations in the top row of Fig. 1 a, the proposed stochastic model assumes that there is a consistent virtual graph that captures the coherent root modular structure. As a specific application of our approach, we can think of multiple networks profiled with different techniques or characterized by multiple types of interactions between vertices. Figure 1 b shows the graphical representation of our extended SBM from traditional SBM for analyzing multiple noisy networks. In our model, every observed network *A* ^(*t*)^ is associated with a latent modular structure $\vec {z}^{(t)}$. These instantaneous structures are considered as the results from stochastic transitions from a latent root modular structure $\vec {z}$ that is coherent in all networks. Note that this is in contrast to previous dynamic models that concentrate on the evolution of modular structures rather than embedding them. The probabilistic inference task is to *simultaneously* learn the root as well as instantaneous modular structures from multiple noisy networks. With such a probabilistic model, we are able to elicit the essential modular structure in all the observed networks. By combining information from these various sources, we can compensate for the perturbation effect from noisy observations. To infer this extended SBM for multiple-network clustering, a variational Bayes method is derived to efficiently quantify uncertainties over unknown model parameters. We apply our method to protein-protein interaction (PPI) data sets and show that by taking advantage of different sources of information, our method outperforms the existing SBM-based methods implemented on individual networks in predicting new protein complexes. Furthermore, the capability of predicting missing edges from our Bayesian modeling creates the opportunity for our method to be used in active learning scenarios, where the task is to efficiently infer protein-protein interactions from new sets of experiments and concurrently by taking advantage of prior knowledge from the existing experimental results, which the individual network SBM lacks.

## Background

We first briefly review the stochastic block model for module identification in individual networks to study the modular structures [8], for which a Bayesian module identification algorithm has recently been proposed to efficiently solve the problem [9].

Given a network in a graph representation, *G*=(*V, E*), where *V* denotes the set of all *N* vertices in the given network and *E* is the set of edges connecting the corresponding vertices in the network *G*. Let *A* be the observed *N*×*N* adjacency matrix whose elements take the values 0 or 1: *A*_*ij*_=1 indicating that there is a corresponding edge *e*_*ij*_∈*E* between vertices *v*_*i*_ and *v*_*j*_∈*V* and *A*_*ij*_=0 otherwise. We introduce the latent variable *z*_*i*_∈{1,2,…,*K*} to represent the module assignment of vertices *v*_*i*_, and *K* is the total number of desirable modules. In SBM, the probability that an edge exists between two vertices depends on their module memberships. Conditioning on module assignments, the probabilities that corresponding vertices are linked follow Bernoulli distributions with the corresponding bias parameters *θ*_*c*_=*p*(*A*_*ij*_=1|*z*_*i*_=*z*_*j*_) and *θ*_*d*_=*p*(*A*_*ij*_=1|*z*_*i*_≠*z*_*j*_), which are called within- and between-module edge probabilities, respectively. Also, SBM assumes a multinomial distribution over module assignment probabilities with parameters $\pi _{k}=p(z_{i}=k| \vec {\pi })$. With these assumptions, the joint probability of an adjacency matrix *A* and the corresponding module assignment vector $\vec {z}$ can be written as

(1) $$\begin{array}{@{}rcl@{}} p\left(A,\vec{z}|\vec{\theta},\vec{\pi},K \right) &=& p\left(A|\vec{z},\vec{\theta}\right)p\left(\vec{z}|\vec{\pi}\vphantom{\dot{\vec{\theta}}\!}\right) \\&=& \theta_{c}^{c^{+}} \left(1-\theta_{c}\right)^{c^{-}} \theta_{d}^{d^{+}} (1-\theta_{d})^{d^{-}} \prod_{k=1}^{K} \pi_{k}^{n_{k}}, \end{array} $$

in which $c^{+}=\sum _{i>j}A_{\textit {ij}} I\left [z_{i}=z_{j}\right ]$ is the number of edges contained within potential modules; $c^{-}=\sum _{i>j}(1-A_{\textit {ij}}) I\left [z_{i}= z_{j}\right ]$ is the number of non-edges contained within modules; $d^{+}=\sum _{i>j}A_{\textit {ij}} I\left [z_{i} \neq z_{j}\right ]$ is the number of edges between vertices across different modules; $d^{-}=\sum _{i>j}(1-A_{\textit {ij}}) I\left [z_{i} \neq z_{j}\right ]$ is the number of non-edges across potential modules; and *n*_*k*_ denotes the number of vertices assigned to the *k*th potential module with $\sum _{k} n_{k} =N$. *I*[ *x*] denotes the indicator function, which equals to one if its argument *x* is a true logic statement and zero otherwise. The factorization of the joint probability follows from the fact that the probability of the observed adjacency matrix can be completely determined based on the given model parameters, including module assignment probabilities $\vec {\pi }$ and within- and between-module edge probabilities *θ*_*c*_, *θ*_*d*_.

## Methods

We extend the above Bayesian framework for individual networks to more robust and accurate module identification across multiple networks. A variational Bayes approach is then derived to infer the unknown parameters of our extended model to identify significant modules across multiple noisy networks.

### Multiple-network stochastic block model

Given multiple observed noisy networks with corresponding adjacency matrices {*A* ^(1)^,*A* ^(2)^,…,*A* ^(*T*)^}, we aim to study the hidden modular structures across these networks. Without loss of generality, we assume that the set of vertices is fixed in all adjacency matrices. To infer the modular structures of these observed networks, we introduce a latent root module assignment $\vec {z}$, which can be considered to determine the connectivity of a virtual image graph illustrated in Fig. 1. For *T* observed networks, the corresponding instantaneous module assignments $\vec {z}^{t}$ for *A* ^(*t*)^ evolve under a transition probability matrix *P* ^(*t*)^. This model allows an inherent modular structure to unify all other observations to borrow strengths from each other when inferring modules of a certain network and thereby compensates for the potential detrimental effect of noise mixed with observations.

With the underlying assumption that multiple observed networks have modular structures with similar within- and between-module edge densities, we fix the edge probabilities *θ*_*c*_ and *θ*_*d*_ to be the same for all the observed networks. To fully specify this new stochastic block model, we set the root assignment matrix $\vec {z}$ to be multinomial with assignment probabilities $\vec {\pi }$. We can write the joint distribution of assignment matrices and observed adjacency matrices of this model as follows:

(2) $$  \begin{aligned} &p\left(A^{(1:T)},\vec{z},\vec{z}^{(1:T)}|\vec{\theta},\vec{\pi},P^{(1:T)},K \right) \\ &\quad = \left[ \prod_{t=1}^{T} p\left(A^{(t)}|\vec{z}^{(t)},\vec{\theta}\right) p\left(\vec{z}^{(t)}|\vec{z},P^{(t)}\right) \right] p(\vec{z}|\vec{\pi}) \\ &\quad = \theta_{c}^{\sum_{t=1}^{T}c_{t}^{+}} (1-\theta_{c})^{\sum_{t=1}^{T}c_{t}^{-}} \theta_{d}^{\sum_{t=1}^{T}d_{t}^{+}} (1-\theta_{d})^{\sum_{t=1}^{T}d_{t}^{-}} \\ & \qquad\times \left[\prod_{t=1}^{T} \prod_{i=1}^{N} \prod_{r,s=1}^{K} P_{rs}^{I\left[z_{i}=r\vphantom{\dot{z_{K_{d}}}\!}\right]\cdot I\left[z_{i}^{(t)}=s\right]} \right] \prod_{k=1}^{K} \pi_{k}^{n_{k}}, \end{aligned}  $$

where a concise index representation (1:*T*) is adopted to denote the indices of the corresponding components in the model for multiple networks. For example, *A* ^(1:*T*)^ stands for *T* adjacency matrices {*A* ^(1)^,…,*A* ^(*T*)^}. The corresponding numbers of edges $c_{t}^{+}$, $c_{t}^{-}$, $d_{t}^{+}$, and $d_{t}^{-}$ for the *t*th network are defined similarly as in the model (1) for individual networks, except that the adjacency matrix *A* is replaced with *A* ^(*t*)^. Similarly, $I\left [z_{i}=r\vphantom {\dot {z_{i}^{(t)}}\!}\right ]\cdot I\left [z_{i}^{(t)}=s\right ]$ counts the vertice *v*_*i*_ when it is assigned to the *s*th module for the *t*th network and in the *r*th module in the root assignment; *n*_*k*_ is calculated from the root assignment. One immediate consequence of such modeling is that the edges that frequently appear in multiple observations have a higher chance of being true positives. Such an intuition is reflected in the likelihood function in our model. In addition, the model makes sure that the vertices connected by these edges are more likely to be assigned to the same modules in different observed networks by the proper choice of transition probabilities, which is clarified in the subsequent section.

### Bayesian inference

To predict module assignments to assign memberships to all the vertices in the given networks, we resort to Bayesian inference to draw a joint posterior distribution of all the latent variables and unknown model parameters. To facilitate the computation of the posterior, we prefer more efficient variational Bayes algorithms instead of directly implementing Monte Carlo (MC) simulations. In order to derive closed-form updates for variational Bayes algorithms, we adopt conjugate prior distributions in our multiple-network clustering model. The conjugate prior for the root assignment probability distribution $\vec {\pi }$ is a Dirichlet distribution with a hyper-parameter vector $\vec {n_{0}}$:

(3) $$\begin{array}{@{}rcl@{}} p\left(\vec{\pi}|\vec{n}_{0}\right)=\frac{\Gamma \left(\sum_{k=1}^{K} n_{k,0}\right)}{\prod_{k=1}^{K}\Gamma (n_{k,0})} \prod_{k=1}^{K} \pi_{k}^{n_{k,0}-1}. \end{array} $$

Here *n*_*k*,0_ is the *k*th component of vector $\vec {n_{0}}$ and *Γ*(·) is the gamma function. The conjugate priors for edge weights *θ*_*c*_ and *θ*_*d*_ are beta distributions with hyper-parameters (*α*_*c*,0_,*β*_*c*,0_) and (*α*_*d*,0_,*β*_*d*,0_), respectively,

(4) $$ {\fontsize{8.9}{6}\begin{aligned} &p\left(\vec{\theta}|\vec{\alpha}_{0},\vec{\beta}_{0}\right) = p(\theta_{c}|\alpha_{c,0},\beta_{c,0})p(\theta_{d}|\alpha_{d,0},\beta_{d,0})\\ &\quad= \frac{\Gamma (\alpha_{c,0} + \beta_{c,0})} {\Gamma (\alpha_{c,0}) \Gamma (\beta_{c,0})} \theta_{c}^{\alpha_{c,0}-1} (1-\theta_{c})^{\beta_{c,0} -1}\\ &\qquad\times \frac{\Gamma (\alpha_{d,0} + \beta_{d,0})} {\Gamma (\alpha_{d,0}) \Gamma (\beta_{d,0})} \theta_{d}^{\alpha_{d,0}-1} (1-\theta_{d})^{\beta_{d,0} -1}. \end{aligned}}  $$

The underlying assumption here is that prior to observing the data, within- and between-module edge weights are independent, so their joint prior distribution factorizes. The transition probability matrices *P* ^(*t*)^ are stochastic, and therefore, their rows add up to 1. For each matrix *P* ^(*t*)^, where *t*∈{1,2,…,*T*}, we use Dirichlet prior distributions with a hyper-parameter vector $\vec {\eta }_{k}^{(0)}$ on rows

(5) $$ {\fontsize{8.6}{6}\begin{aligned} p\left(P^{(t)}|\vec{\eta}_{1}^{(0)},\ldots,\vec{\eta}_{K}^{(0)}\right) &= \prod_{k=1}^{K} p\left(\vec{P}_{k}^{(t)}|\vec{\eta}_{k}^{(0)}\right) \\ &= \prod_{k=1}^{K} \frac{\Gamma \left(\sum_{m=1}^{K} \eta_{k,m}^{(0)}\right)} {\prod_{m=1}^{K}\Gamma \left(\eta_{k,m}^{(0)}\right)} \times \prod_{m=1}^{K} \left(P_{km}^{(t)}\right)^{\eta_{k,m}^{(0)}-1}, \end{aligned}}  $$

where $\vec {P}_{k}^{(t)}$ is the *k*th row of the transition probability matrix *P* ^(*t*)^, $P_{\textit {km}}^{(t)}$ is its *m*th element, and $\eta _{k,m}^{(0)}$ is the *m*th element of $\vec {\eta }_{k}^{(0)}$. The rows of transition probability matrices are assumed to be independent, and also, we set their hyper-parameter vectors to be identical.

To further ensure that our model captures the modular structure inherent in the observed networks, we set hyper-parameters of prior beta distributions over edge weights to bias towards edge weights with within-module edge weights being greater than between-module edge weights, and this is controlled through appropriate settings of hyper-parameters of prior beta distributions over edge weights. For the model to be capable of benefiting from the structural information inferred from other networks, we prefer that the diagonal entries of transition probability matrices *P* ^(*t*)^ to be higher than the off-diagonal entries of those matrices, which can be achieved by setting higher hyper-parameters in the corresponding Dirichlet distributions.

With these incorporated conjugate priors, their functional forms are preserved in the posterior, a variational Bayes algorithm with closed-form updates can be derived to infer the model parameters, and, more importantly, module memberships from the aforementioned model (2) in the subsequent section.

### Variational Bayes solution

Variational Bayes method is an efficient alternative to Monte Carlo sampling methods [16, 17] for statistical inference over complicated models as direct sampling is not tractable and computationally prohibitive. Under appropriate settings, variational Bayes algorithms can be derived to infer the desired posterior distributions with comparable accuracies at a greater speed, which is essential for the analysis of large-scale networks. The variational Bayes method seeks a restricted family of approximation distributions *q*(·), which minimize the Kullback-Leibler (KL) divergence between the joint probability distributions of unknown parameters and their approximate joint probability distributions [18]. For our proposed model, the quantity to be minimized takes the following form:

(6) $$ { \begin{aligned} F\left\{q,A^{(1:T)}\right\} = &- \sum_{\vec{z},\vec{z}^{(1:T)}} \int \int \left[q\left(\vec{z},{\vphantom{{\sum_{0}^{0}}}}\vec{z}^{(1:T)},\vec{\theta},\vec{\pi}\right) \right.\\ & \times \left. \ln \frac{p\left(A^{(1:T)},\vec{z},\vec{z}^{(1:T)},\vec{\theta},\vec{\pi}|K\right)} {q\left(\vec{z},\vec{z}^{(1:T)},\vec{\theta},\vec{\pi}\right)} \right] d \vec{\theta} d \vec{\pi}. \end{aligned}}  $$

To simplify this optimization problem of minimizing the free energy *F*{*q,A* ^(1:*T*)^}, we follow the mean field approximation framework developed in physics [9]. To be specific, we factorize the variational or approximate distribution *q*(·) with respect to its arguments:

(7) $$\begin{array}{@{}rcl@{}} q\left(\vec{z},\vec{z}^{\,\left(1:T\right)},\vec{\theta},\vec{\pi}\right) = q_{\vec{\theta}}(\vec{\theta})q_{\vec{\pi}}(\vec{\pi})q_{\vec{z}}(\vec{z}) \prod_{t=1}^{T} q_{\vec{z}^{(t)}}\left(\vec{z}^{(t)}\right). \end{array} $$

After this simplification, it can be shown that the optimal approximate distribution $q_{\vec {z}}$ for the root module assignment $\vec {z}$ satisfies the following equation [18]:

(8) $$\begin{array}{@{}rcl@{}} \ln q_{\vec{z}}^{*}(\vec{z}) \propto E_{-\vec{z}} \left[\ln p\left(A^{(1:T)},\vec{z},\vec{z}^{(1:T)},\vec{\theta},\vec{\pi}|K\right) \right], \end{array} $$

where $E_{-\vec {z}} [\cdot ]$ denotes the expectation taken over all the parameters and latent variables except $\vec {z}$. Similar equations can be derived for $\vec {\pi }$, $\vec {\theta }$, and $\vec {z}^{(t)}$ for *t*∈{1,2,…,*T*}. Solving the above Eq. (8) for all the unknown parameters leads to the complete derivation of the approximate distributions.

Particularly, these distributions belong to the same family as prior distributions, i.e., the approximate distributions of *θ*_*c*_, *θ*_*d*_, and $\vec {\pi }$ are respectively beta, beta, and Dirichlet distributions with hyper-parameters $\left (\tilde {\alpha }_{c},\tilde {\beta }_{c}\right)$, $\left (\tilde {\alpha }_{d},\tilde {\beta }_{d}\right)$, and $\tilde {\vec {n}}$. In order to calculate the posterior approximate distribution of module assignments, we factorize them as *q*(*z*_*i*_=*k*)=*Q*_*ik*_ and $q\left ({z_{i}^{t}}=k\right)=Q_{\textit {ik}}^{(t)}$ for *i*∈{1,2,…,*N*}, *t*∈{1,2,…,*T*}, and *k*∈{1,2,…,*K*}. *Q* and *Q* ^(*t*)^ are *N*×*K* matrices, in which the *i*th row denotes the probability of assigning vertex *v*_*i*_ to different potential modules.

The variational Bayes algorithm iterates between two stages. In the first step, the current distributions over the model parameters are used to evaluate the module assignment matrices *Q* and *Q* ^(*t*)^; and in the second step, these memberships are fixed and variational distributions over model parameters are updated. The resulting iterative algorithm then can be summarized as:

*Initialization*. Initialize *N*×*K* matrices *Q* and *Q* ^(*t*)^ for *t*∈{1,2,…,*T*} and set $\tilde {\alpha }_{c}=\alpha _{c,0}$, $\tilde {\beta }_{c}=\beta _{c,0}$, $\tilde {\alpha }_{d}=\alpha _{d,0}$, $\tilde {\beta }_{d}=\beta _{d,0}$, and $\tilde {\vec {n}} = \vec {n}_{0}$.

(i) Update the following expected values:
  (9) $$\begin{array}{@{}rcl@{}} E\left[\ln \pi_{k}\right] = \psi(\tilde{n}_{k}) - \psi\left(\sum_{k=1}^{K}\tilde{n}_{k}\right); \end{array} $$
  (10) $$\begin{array}{@{}rcl@{}} E\left[\ln P_{km}^{(t)}\right] = \psi\left(\tilde{\eta}_{k,m}^{(t)}\right) - \psi\left(\sum_{m=1}^{K} \tilde{\eta}_{k,m}^{(t)}\right); \end{array} $$
  (11) $$\begin{array}{@{}rcl@{}} E\left[\ln \frac{1-\theta_{d}}{1-\theta_{c}}\right] &=& \psi\left(\tilde{\beta}_{d}\right) - \psi\left(\tilde{\alpha}_{d}+\tilde{\beta}_{d}\right)- \psi\left(\tilde{\beta}_{c}\right)\\ &&+ \psi\left(\tilde{\alpha}_{c}+\tilde{\beta}_{c}\right); \end{array} $$
  (12) $$\begin{array}{@{}rcl@{}} E\left[ \ln \frac{1-\theta_{d}}{1-\theta_{c}} + \ln \frac{\theta_{c}}{\theta_{d}}\right] &=& \psi(\tilde{\alpha}_{c}) - \psi(\tilde{\beta}_{c})- \psi(\tilde{\alpha}_{d})\\ &&+ \psi(\tilde{\beta}_{d}), \end{array} $$
  where *ψ*(·) is the digamma function.
(ii) Update the variational distribution over the root module assignment:
  (13) $$\begin{array}{@{}rcl@{}}  Q_{ik} \propto \exp \left\{E\left[\ln \pi_{k}\right] + \sum_{t=1}^{T} \sum_{m=1}^{K} Q_{im}^{(t)} E\left[\ln P_{km}^{(t)}\right]\right\}. \end{array} $$
  Normalize *Q* such that $\sum _{k=1}^{K} Q_{\textit {ik}}=1$ for all vertices *v*_*i*_.
(iii) Update the variational distributions over instantaneous module assignments for *t*∈{1,2,…,*T*}:
  (14) $$\begin{array}{@{}rcl@{}}  Q_{ik}^{(t)} &\propto& \exp \left\{\sum_{j \neq i} \left(E\left[\ln \frac{1-\theta_{d}}{1-\theta_{c}} + \ln \frac{\theta_{c}}{\theta_{d}}\right] A_{ij}^{(t)} \right. \right.\\ &-& \!\!\left.\left. E\left[ \ln \frac{1-\theta_{d}}{1-\theta_{c}}\right]\right) Q_{jk}^{(t)} + \sum_{s=1}^{K} Q_{is} \left[\ln P_{sk}^{(t)}\right]\right\}. \end{array} $$
  Normalize *Q* ^(*t*)^ such that $\sum _{k=1}^{K} Q_{\textit {ik}}^{(t)}=1$ for all vertices *v*_*i*_.
(iv) Update the posterior hyper-parameters of the Dirichlet distribution over the root module assignment of vertices:
  (15) $$\begin{array}{@{}rcl@{}} n_{k}=\sum_{i=1}^{N} Q_{ik} + n_{k,0}. \end{array} $$
(v) Consider *η* ^(*t*)^ for *t*∈{1,2,…,*T*} as a matrix whose elements are $\eta _{k,m}^{(t)}$. Then, update the matrix *η* ^(*t*)^ as follows:
  (16) $$\begin{array}{@{}rcl@{}} \eta^{(t)} = Q^{\prime}Q^{(t)} + \eta^{(0)}, \end{array} $$
  where *Q* ^′^ is the transpose of the matrix *Q* and *η* ^(0)^ is the matrix of prior hyper-parameters of transition probability matrices.
(vi) Update the hyper-parameters of beta distributions over edge weights:
  (17) $$\begin{array}{@{}rcl@{}} \tilde{\alpha}_{c} = \frac{1} {2} \sum_{t=1}^{T} Tr\left(Q^{(t)'}A^{(t)}Q^{(t)}\right) + \alpha_{c,0}; \end{array} $$
  (18) $$\begin{array}{@{}rcl@{}} \tilde{\beta}_{c} &=& \frac{1} {2} \sum_{t=1}^{T} Tr\left(Q^{(t)'}\left(\vec{u} \vec{v}^{(t)'}-Q^{(t)}\right)\right) \\&&- \frac{1} {2} \sum_{t=1}^{T} Tr\left(Q^{(t)'}A^{(t)}Q^{(t)}\right)+ \beta_{c,0}; \end{array} $$
  (19) $$\begin{array}{@{}rcl@{}} \tilde{\alpha}_{d} = \sum_{t=1}^{T} \sum_{i>j} A_{ij}^{(t)} - \frac{1} {2} \sum_{t=1}^{T} Tr\left(Q^{(t)'}A^{(t)}Q^{(t)}\right)+ \alpha_{d,0}; \end{array} $$
  (20) $$  \begin{aligned} \tilde{\beta}_{d} &= \sum_{t=1}^{T} \sum_{i>j} \left(1-A_{ij}^{(t)}\right)- \frac{1} {2} \sum_{t=1}^{T} Tr\left(Q^{(t)'}\left(\vec{u} \vec{v}^{(t)'}-Q^{(t)}\right)\right)\\ &\quad + \frac{1} {2} \sum_{t=1}^{T} Tr\left(Q^{(t)'}A^{(t)}Q^{(t)}\right) + \beta_{d,0}, \end{aligned}  $$
  where $\vec {u}$ is a *N*×1 vector of ones and $\vec {v}^{(t)}$ is a vector with elements $v_{k}^{(t)}=\sum _{i=1}^{N} Q_{\textit {ik}}^{(t)}$.
(vii) Calculate the updated free energy:
  (21) $$ {\fontsize{9}{6}\begin{aligned} & F\left\{q^{*},A^{(1:T)}\right\} = \sum_{t=1}^{T} \sum_{i=1}^{N} \sum_{k=1}^{K} Q_{ik}^{(t)} \ln Q_{ik}^{(t)} + \sum_{i=1}^{N} \sum_{k=1}^{K} Q_{ik} \ln Q_{ik}\\ &- \sum_{t=1}^{T} \sum_{k=1}^{K} \ln \frac {B\left(\tilde{\vec{\eta}}_{k}^{(t)}\right)} {B\left(\vec{\eta}_{k}^{(0)}\right)} - \ln \frac {B\left(\tilde{\alpha}_{c},\tilde{\beta}_{c}\right)B\left(\tilde{\alpha}_{d},\tilde{\beta}_{d}\right)B\left(\tilde{\vec{n}}\right)} {B\left(\alpha_{c,0},\beta_{c,0}\right)B\left(\alpha_{d,0},\beta_{d,0}\right)B(\vec{n}_{0})}, \end{aligned}}  $$
  where *B*(·) is a beta function with the vector argument.

The optimized free energy in (21) decreases in consecutive iterations, and thereby, this algorithm is guaranteed to converge to a local optimum. In the case where the posterior is multi-modal, several initializations should be tested to ensure the quality of the returned solutions.

## Experimental results

In this section, we evaluate our Bayesian module identification across multiple networks by testing the derived variational Bayes algorithm on both synthetic and real-world PPI data sets. The obtained results also are compared with Bayesian module identification with individual networks and another state-of-the-art network clustering algorithm—ClusterOne [19]. For synthetic data, we have the ground-truth module memberships and therefore we use normalized mutual information to assess the performance of our model. Normalized mutual information (NMI) is defined as follows [3, 13]:

(22) $$\begin{array}{@{}rcl@{}} \text{NMI}(\mathcal{C,C'})=\frac{\hat{\text{MI}}\left(\mathcal{C,C'}\right)}{\text{max}(H(\mathcal{C}),H(\mathcal{C'}))}, \end{array} $$

where $\mathcal {C}=\{C_{1},C_{2},\ldots,C_{K}\}$ denotes the true assignments of vertices to corresponding modules and $\mathcal {C'}=\left \{C_{1}',C_{2}',\ldots,C_{K}'\right \}$ denotes the inferred module memberships of vertices by the implemented algorithms. $H(\mathcal {C})$ and $H(\mathcal {C'})$ are the entropies of the ground truth and inferred modules. $\hat {\text {MI}}(\mathcal {C,C'})$ is the mutual information calculated by $\hat {\text {MI}}(\mathcal {C,C'})=\sum _{C_{i},C_{j}'} p\left (C_{i},C_{j}'\right) \ln \frac {p\left (C_{i},C_{j}'\right)}{p\left (C_{i}\vphantom {\dot {C_{j}'}\!}\right)p\left (C_{j}'\right)}$.

For real-world data sets, we analyze two budding yeast (*Saccharomyces cerevisiae*) PPI networks obtained from the Database of Interaction Proteins (DIP) [14] and the Biological General Repository for Interaction Datasets (BioGRID) [15] to predict protein complexes. The *predicted protein complexes* as inferred modules by the selected algorithms are then verified against the Saccharomyces Genome Database (SGD) [20] and Munich Information Center for Protein Sequences (MIPS) [21] golden standards as the *reference complexes*. To validate the predicted protein complexes by the selected algorithms, we adopt the same performance metrics introduced in [3, 19]. The first metric is the fraction of pairs between the predicted and reference complexes with an overlap score of larger than 0.25. We represent this metric in the results with Frac. The overlap score *ω* between two sets of vertices, proteins in this case, *V*_1_ and *V*_2_, is defined as:

(23) $$\begin{array}{@{}rcl@{}} \omega (V_{1},V_{2})= \frac{|V_{1} \cap V_{2}|^{2}}{|V_{1}| |V_{2}|}, \end{array} $$

where |·| denotes the cardinality of a set. The threshold 0.25 used for *ω* is achieved when two equally sized protein complexes have an intersection set with half of their size.

The second performance metric is the geometric accuracy (Acc), which is the geometric mean of two measures: the module-wise sensitivity (Sn) and module-wise positive predictive value (PPV): Acc$~=~\sqrt {\text {PPV} \times \text {Sn}}$. Given *n* reference and *m* predicted protein complexes, *t*_*ij*_ denotes the number of proteins that are the members of both the reference complex *i*: 1≤*i*≤*n* and predicted complex *j*: 1≤*j*≤*m*. Furthermore, let *n*_*i*_ represent the total number of proteins in the reference complex *i*. The two measures Sn and PPV for computing the geometric accuracy are defined as:

$$\begin{array}{@{}rcl@{}} \text{Sn}=\frac{\sum_{i=1}^{n} \max_{j \in \{1,\ldots,m\} }t_{ij}}{\sum_{i=1}^{n} n_{i}}; \end{array} $$

$$\begin{array}{@{}rcl@{}} \text{PPV}= \frac{\sum_{j=1}^{m} \max_{i \in \{1,\ldots,n\} }t_{ij}}{\sum_{j=1}^{m} \sum_{i=1}^{n} t_{ij} }. \end{array} $$

Since Sn can be maximized by putting every protein in the same module and PPV can be maximized by assigning each protein in a distinct module, the Acc is considered a better performance metric that we adopt.

When some proteins appear in either none of the predicted complexes or in more than one complexes, the value of PPV can be misleading [3, 19]. To mitigate such deficiencies, the authors in [22] have introduced an additional metric called module-wise separation (Sep) for fair comparison. To define it, we first introduce the marginal row-wise and column-wise relative frequencies that can be computed as:

$$\begin{array}{@{}rcl@{}} F_{ij}^{r} = \frac{t_{ij}}{\sum_{j=1}^{m} t_{ij}}; \\ F_{ij}^{c} = \frac{t_{ij}}{\sum_{i=1}^{n} t_{ij}}. \end{array} $$

The separation of the predicted complex *i* and reference complex *j* then equals to $\text {Sep}_{\textit {ij}}=F_{\textit {ij}}^{r} F_{\textit {ij}}^{c}$. The reference-wise and inferred-module-wise scores are calculated for the whole set of the reference and predicted complexes as:

$$\begin{array}{@{}rcl@{}} \text{Sep}_{\text{ref}} = \frac{\sum_{i=1}^{n} \sum_{j=1}^{m} \text{Sep}_{ij}}{m}; \\ \mathrm{Sep_{inf}} = \frac{\sum_{i=1}^{n} \sum_{j=1}^{m} \text{Sep}_{ij}}{n}. \end{array} $$

The final separation score is computed from these two quantities as $\text {Sep}=\sqrt {\mathrm {Sep_{\textit {ref}}Sep_{\textit {inf}}}}$. Sep_*ij*_=1 indicates that the reference complex *j* is a perfect match for predicted complex *i* and both of them contain identical proteins.

### Synthetic networks

Using the same procedure as in [1], we generate a synthetic network with *N*=128 vertices and *K*=4 modules, each module containing 32 vertices. The average degree of vertices is set to 16, and the average number of between-module edges of each vertex is set to 6. To generate the network, we first assign vertices to different modules by following a multinomial distribution with the equal weights for all modules. Then, each pair of vertices are connected with the probabilities equal to *θ*_*c*_ or *θ*_*d*_ if they belong to the same or different modules, respectively.

To simulate multiple observed noisy networks, we implement the Sneppen and Maslov re-wiring method [23] to construct new networks and adjacency matrices with instilled noise based on the original network generated as described above. In this method, a pair of edges *v*_*i*_⇔*v*_*j*_ and *v*_*k*_⇔*v*_*ℓ*_ are randomly selected and then re-wired such that *v*_*i*_ becomes connected to *v*_*ℓ*_, while *v*_*j*_ to *v*_*k*_, provided that none of these edges existed previously. This method preserves the degree of each vertex and thus global topological properties, including edge densities in perturbed networks, do not change significantly.

**Fig. 2:**
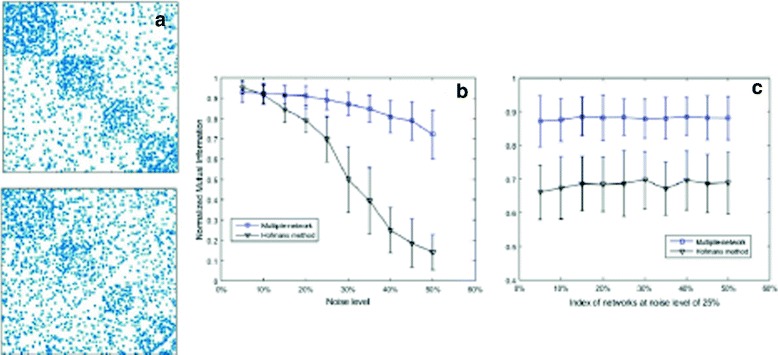
Performance evaluation with synthetic networks. **a** Examples of noisy networks: The top network is with the lowest noise level (5 %) and the bottom network is with the highest noise level (50 %). **b** Performance results of the first set of experiments, where the instilled noise level increases gradually. **c** Performance results of the second set of experiments, where the noise level remains constant

Following this procedure, we generate two different sets of simulated networks. In the first experiment, we generate *T*=10 adjacency matrices where the number of randomly selected re-wirings increases linearly from 5 to 50 % of the total number of edges in nine steps gradually. The adjacency matrices for two extreme cases at *t*=1 and *t*=10 are shown in Fig. 2 a respectively on top and bottom rows, which reflect different levels of introduced noise. In the second experiment, *T*=10 adjacency matrices are generated from an original adjacency matrix by randomly re-wiring 25 % of the total number of edges. Thereby, here, the noise levels are consistent across all the networks. Note that the re-wirings are independent from each other. Intuitively, in the first set of networks, module identification becomes more difficult with increasing noise levels while in the second experiment, it is similarly difficult when identifying modules in respective networks.

To demonstrate that our Bayesian module identification across multiple networks can better identify modules by borrowing strength across networks, we compare the results by our algorithm on the set of ten randomly perturbed networks with those of Hofman’s algorithm [9] applied to individual networks. Since we assume no prior knowledge on module memberships of the vertices, the initial hyper-parameters for the Dirichlet distributions for module assignments are set to equal values for all *K*=4 modules. Empirically, neither of the algorithms is sensitive to hyper-parameters for beta distributions over edge weights, provided that within-module edge weights are larger than the between-module counterpart. Based on our experiments, Hofman’s implementation on individual networks may not converge to the global optimum, especially when we have high levels of introduced noise, for example, when we have 20 % random re-wirings (*t*>4) in our experiments. On the contrast, we find that multiple random initializations may not be necessary for our multiple-network clustering algorithm. In order to have a fair comparison with the satisfactory solution quality, we take 100 random initializations for both algorithms.

Figure 2 b and c depict the average normalized mutual information in two experiments between module detection results of both algorithms and the true module membership, by which data has been generated, based on averaged 100 independent repeats with the aforementioned settings. As it can be seen in these figures, as the noise level increases, the difference between the performances of two algorithms gets more significant. For highly noisy adjacency matrices, Hofman’s algorithm indeed fails to recover the module memberships accurately. Nonetheless, our algorithm by borrowing information from other observations returns satisfying results. In the second experiment, we can clearly observe the superiority of our model as there is an approximate 0.2 difference in the normalized mutual information measure in favor of our algorithm across all networks. Thus, aggregating information across networks has led to higher accuracy in predicting the module membership of vertices achieved by our method.

### Edge prediction

To further validate our model, we simulate two networks from a single “ground truth” network with the specifications identical to the networks generated in the previous section, by instilling 25 % noisy edges based on the Sneppen and Maslov re-wiring method. To test the capability of our model to predict missing edges of the ground truth network, we randomly hold out different percentages of edges of the first network and use the remaining edges (and the other network in the case of our multiple-network model) to predict the probability of each missing edge to exist. Compared to previous experiments, we only need to slightly modify the inference by discarding the held-out edges from the likelihood. The probability of an edge existing between nodes *i* and *j* in the model can be calculated by

$$\begin{array}{@{}rcl@{}} p(A_{ij}\,=\,1)\,=\,\left(\frac{\tilde{\alpha}_{c}}{\tilde{\alpha}_{c} +\tilde{\beta}_{c}}-\frac{\tilde{\alpha}_{d}}{\tilde{\alpha}_{d}+\tilde{\beta}_{d}} \right) \sum_{k=1}^{K} Q_{ik}^{(t)}Q_{jk}^{(t)}\,+\, \frac{\tilde{\alpha}_{d}}{\tilde{\alpha}_{d} +\tilde{\beta}_{d}}. \end{array} $$

**Fig. 3:**
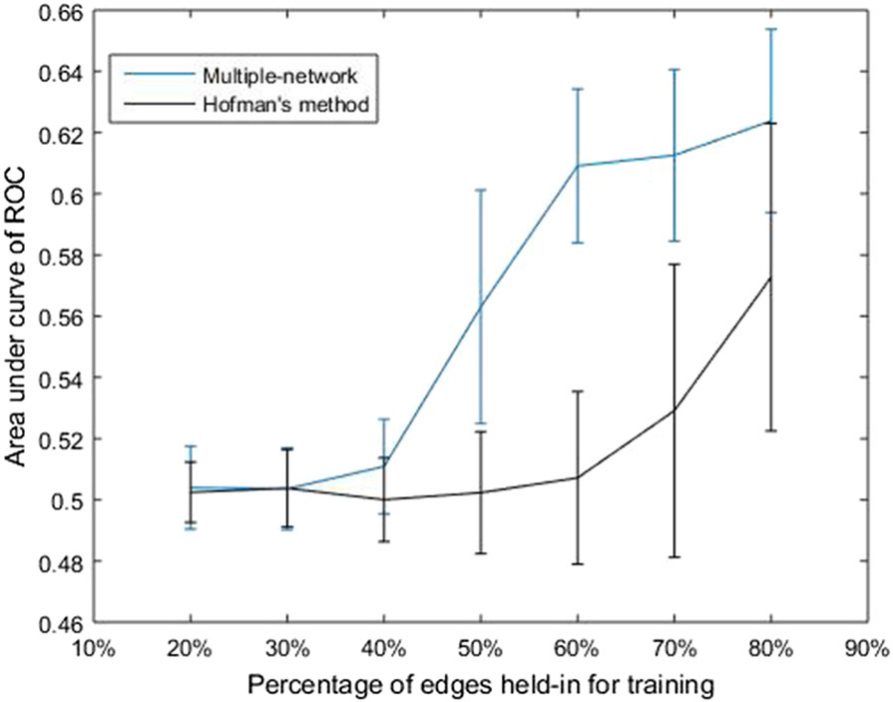
Error bar plots of Area Under the Curve (AUC) of ROC for training ratios ranging from 20 to 80 %

**Fig. 4:**
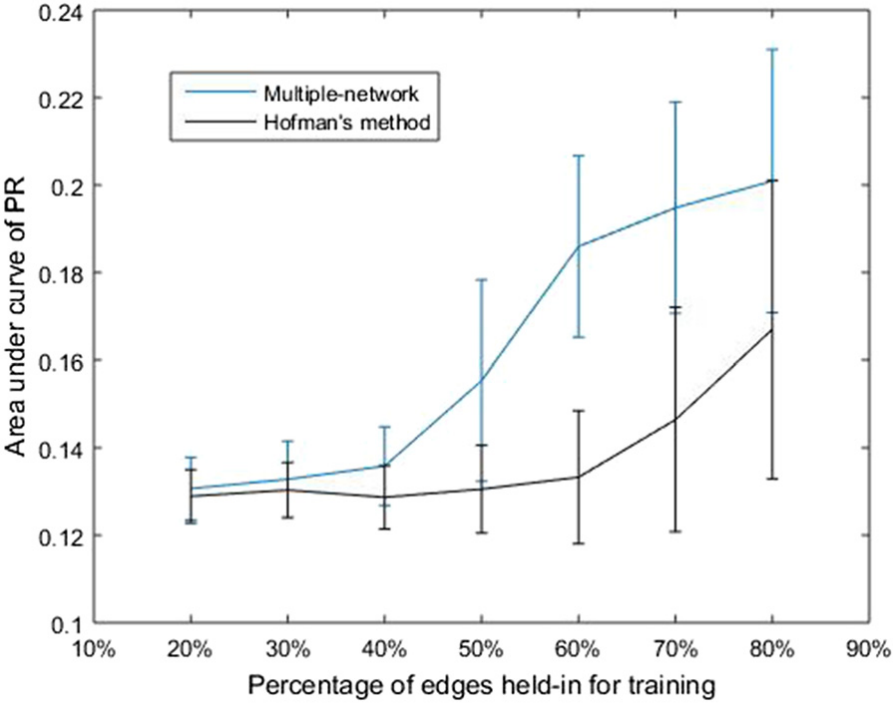
Error bar plots of Area Under the Curve (AUC) of Precision-recall (PR) for training ratios ranging from 20 to 80 %

We consider different training ratios (the percentage of remaining edges in one network) ranging from 20 to 80 % with 10 % increments. Figures 3 and 4 show the error bar plots of Area Under the Curves (AUC) of both the Receiver Operating Characteristic (ROC) and Precision-recall (PR) for different training ratios, respectively. As expected, our multiple-network method outperforms Hofman’s model in terms of both ROC and PR as it takes advantage of the shared information across observations. As shown in Figs. 3 and 4, decreasing the number of held-out edges leads to the growing margin between the performances of the two approaches.

### Protein complex prediction

We further have applied our Bayesian module identification to unweighted yeast PPI networks, extracted from DIP and BioGrid, to predict protein complexes. Each of these networks have 4540 proteins and the number of edges in DIP and BioGrid networks are 21,326 and 49,128, respectively. Besides our algorithm, ClusterOne [19] and Hofman’s method [9] also have been applied to the networks for comparison. ClusterOne is a greedy algorithm that can be considered as an overlapping extension of normalized cut spectral clustering. For both Hofman’s and our algorithm, we need to decide the value of *K* for the number of potential modules. However, we note that both algorithms are in the Bayesian framework and thereby the full probability of memberships of different modules can be determined. With the large enough *K*, model likelihoods for different *K*s can be evaluated to determine the optimal *K*. In the current experiments, we also focus on non-overlapping module identification as done in [9] for fair comparison by assigning each vertex *v*_*i*_ to the *k*th module that maximizes $Q_{\textit {ik}}^{(t)}$ in the *t*th network. Based on the average size of protein complexes given in yeast golden standards, which is approximately 5 in both SGD and MIPS, we set *K*=1000 considering the size of our PPI networks.

The results of these three methods are compared based on the parameters introduced earlier. Table 1 contains the performance comparison between these algorithms based on the SGD golden standard while Table 2 provides the performance comparison based on the MIPS standard. One advantage of Bayesian methods is that the identified complexes cover the whole set of proteins present in the data sets, while ClusterOne only discovers overlapping complexes that include 1372 and 2340 proteins for DIP and BioGRID datasets respectively out of the total 4540 proteins in the original networks. As a consequence, complexes found by Bayesian methods have larger sizes and therefore possess lower fraction of matched proteins compared to the reference complexes, which are reflected as low PPV values in both tables. Nonetheless, the results with respect to the Acc have shown that our new Bayesian module identification with multiple networks can better predict potential protein complexes, in which proteins are densely connected; and thereby, these methods are more useful when the final objective is to predict new protein complexes. Another observation in the results is that combining information from different observed networks in our model enhances both the Acc and Sep metrics compared with ClusterOne and Hofman’s method, especially for the DIP network, a relatively sparse network, for which detecting inherent modules is a difficult task. Table 3 depicts the number of predicted complexes for different data sets by different algorithms.

**Table 1:** Performance comparison of different algorithms based on SGD golden standard

Data set	Metric	Multiple network	ClusterOne	Hofman
DIP	Acc	**0.5435**	0.4731	0.4561
	Frac	0.2129	**0.3194**	0.1000
	PPV	0.4648	**0.5528**	0.3295
	Sep	**0.3511**	0.3329	0.3146
BioGRID	Acc	**0.6110**	0.5961	0.5549
	Frac	0.2097	**0.4839**	0.1871
	PPV	0.4738	**0.5663**	0.4612
	Sep	**0.3999**	0.3325	0.3505

The best indices are highlighted with bold fonts

**Table 2:** Performance comparison of different algorithms based on MIPS golden standard

Data set	Metric	Multiple network	ClusterOne	Hofman
DIP	Acc	**0.3933**	0.3178	0.3403
	Frac	0.2381	**0.3598**	0.1111
	PPV	0.3567	**0.4076**	0.2651
	Sep	**0.2535**	0.2216	0.2020
BioGRID	Acc	**0.4614**	0.4336	0.4383
	Frac	0.2975	**0.4974**	0.2275
	PPV	0.3713	**0.4207**	0.3649
	Sep	**0.2536**	0.2193	0.2189

The best indices are highlighted with bold fonts

**Table 3:** Number of identified protein complexes by different algorithms for DIP and BioGRID data sets

Data set	Multiple network	ClusterOne	Hofman
DIP	320	328	112
BioGRID	278	424	189

**Fig. 5:**
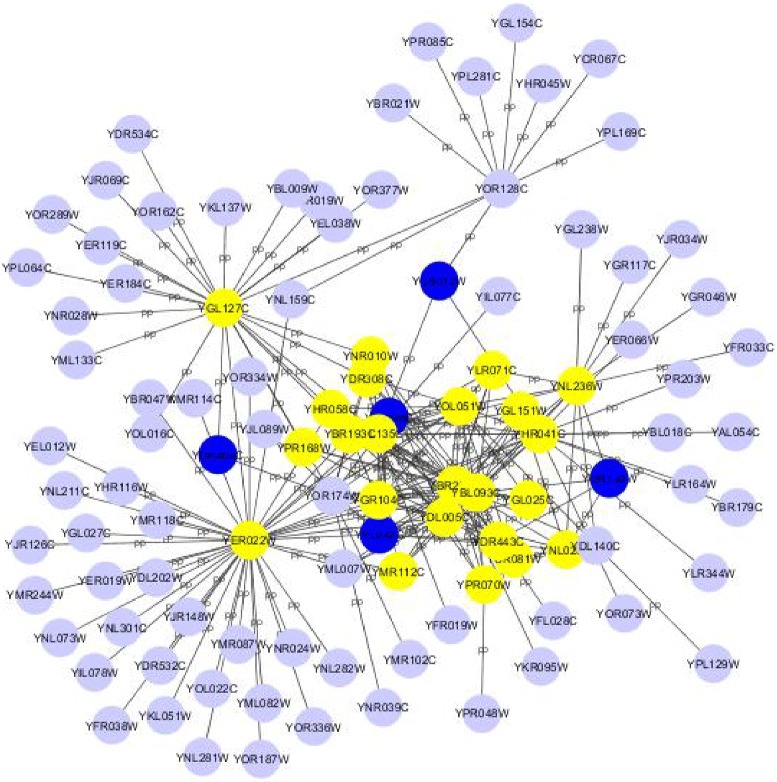
One example of the predicted protein complexes by Hofman’s and our multiple-network clustering algorithms. The whole set of proteins were considered as a single complex by Hofman’s method, while the proteins colored in *yellow* and *dark blue* form the predicted complex returned by our new multiple-network clustering method. The colored proteins in *yellow* are the member proteins in the RNA polymerase II mediator protein complex in SGD golden standard. This figure is produced by [24]

It is clear that, compared to its individual-network counterpart—Hofman’s method—our algorithm is able to find significantly more protein complexes and thus obtaining a better understanding of both networks’ modular structures. One example is illustrated in Fig. 5. This figure demonstrates the interactions among a group of proteins in the DIP data set. All of these proteins are assigned to the same complex by Hofman’s method. However, our multiple-network method identified a more densely connected subset of proteins. This discovered protein complex contains all of the proteins in the reference RNA polymerase II mediator protein complex in SGD golden standard. These proteins are colored in yellow in the figure, and other proteins that do not belong to this reference complex but are members of the predicted complex by our algorithm are colored in blue. Thus, we can observe that our method is capable of increasing the resolution in module identification. Note that the number of modules found by our algorithm is comparable to that of ClusterOne, though the modules in our method are disjoint. Since we have the posterior distribution of module assignments of all vertices, one can easily construct lots of overlapping complexes by allowing each vertex to belong to more than one modules.

## Conclusions

In this paper, we generalize the variational Bayes algorithm for module identification in individual networks [9] to a new stochastic block model with the efficient accompanying variational Bayes algorithm for module identification across multiple noisy observed networks. The effectiveness and efficiency of our algorithm with improved accuracy and resolution have been demonstrated on both synthetic and real-world PPI networks. In our future work, we will focus on finding solution methods for module identification from multiple networks with more general noise models.
